# Unintentional firearm deaths among children, 0–17 years of age, by race: Findings from the national violent death reporting system, 2015–2021

**DOI:** 10.1186/s40621-025-00573-1

**Published:** 2025-05-02

**Authors:** Samuel Fischer, Matthew Miller, Eliot W. Nelson, Christopher Chang, Deborah Azrael

**Affiliations:** 1 Harvard Injury Control Research Center, Harvard T. H. Chan School of Public Health, Boston, MA USA; 2 Department of Pediatrics, Larner College of Medicine, University of Vermont, Burlington, VT USA; 3 The Warren Alpert Medical School of Brown University, Providence, Rhode Island USA; 4 Bouvé College of Health Sciences, Department of Public Health and Health Sciences, Northeastern University, 360 Huntington Avenue, Boston, MA 02115 - 5000 USA

**Keywords:** Adolescent, Cause of death, Child, Firearms, Injury, Pediatric, Population surveillance, Unintentional, Violence

## Abstract

**Background:**

Unintentional firearm death (UFD) rates are higher among Black children than among White and Hispanic children. Whether disparities in UFD rates among Black as compared to White and Hispanic children vary by other demographic characteristics or by circumstances is unknown.

**Methods:**

Data come from the 32 states contributing to the National Violent Death Reporting System (NVDRS), 2015–2021. Our sample comprises children 0–17 who died from unintentional firearm injuries. Race/ethnicity- and age-specific population data at the state and county level were used to calculate rates. UFD rates were compared within and across race-ethnicity groupings by age, sex, urbanization and across four NVDRS coded circumstances. Urbanization was assigned using a six-level urban–rural classification scheme from the National Center for Health Statistics (NCHS) based on the county in which the fatal injury occured.

**Findings:**

Of the 568 UFDs, four-fifths of victims were male (82%) and four-fifths died in a home (84%), usually the Victim’s home (55%). Most deaths involved a child playing with a firearm (63%). Overall, UFD rates were 4.6-fold higher for Black children compared with White children. Black children’s rates were more than 6-fold higher than those of White children for females and for children five to nine years of age, and nearly 8-fold higher for children living in large central metro counties.

**Conclusions:**

Black children die from unintentional firearm injury at disproportionately high rates, especially young children living in urban centers. The underlying reasons for these racial disparities are unclear and should be a priority for future research.

## Background

Recent epidemiological studies of unintentional firearm death (UFD) in children have reported striking racial disparities between White and Black children, who together account for more than 80% of these deaths [1–7]. These racial disparities remain incompletely understood, in part because only two of the studies [2, 3] used data from the National Violent Death Reporting System (NVDRS), which has been shown [8–10] to code UFDs more accurately than does the National Vital Statistics System used in earlier studies. Although the studies by Wilson et al., and Vaishnav et al., did not focus on racial rate disparities, both reported that approximately the same number of Black and White children died from UFDs, and thus present NVDRS data indicating that Black children were likely dying at disproportionately elevated rates relative to White children (given the relative size of the respective at-risk populations). Little more is known about racial disparities in UFDs., including whether disparities are accentuated or attenuated by age, sex, or urbanization.

The current study helps fill this research gap using data from states participating in the NVDRS, 2015–2021. Specifically, after linking UFD mortality data for children 0–17 years of age to denominator information about the size of the population at risk, we estimated the relative incidence of UFDs by race/ethnicity, sex, age and urbanization, and compare the circumstances surrounding these deaths by race/ethnicity, including contextual information about where the injury occurred, the relationship of the shooter to the victim, and the type of firearm used.

## Methods

We used data from the NVDRS restricted access dataset. NVDRS is a state-based surveillance system that compiles information from death certificates, law enforcement reports, and coroner/medical examiner reports into an incident-level database, using a trained abstractor [2]. Our analytic sample comprised the 32 states that were funded to contribute data for either 6 or 7 years of the 7-year study period, 2015–2021. UFDs were identified for decedents 0–17 years of age using the abstractor manner of death variable.

Demographic characteristics of decedents, including age, race/ethnicity, and sex were drawn from the NVDRS dataset, as were incident-related descriptive data including type of firearm. Narrative review was used as needed to recover data about the shooter, circumstance, and injury location.

We used National Center for Health Statistics (NCHS) rural/urban classification codes to characterize the urbanicity of the county where the decedent was injured. For this study, we considered counties classified as micropolitan or noncore as “rural,” and all others as “urban.” Some NVDRS racial/ethnic groups—Asian, Two or More Races, and Native American/Alaskan Native—had such low counts of UFDs that data use agreements would have required suppressing nearly all pertinent data for these distinct racial categories. As such, we aggregated these racial groups into an “Other” racial category.

Data regarding circumstances of each death, such as activity or behavior at the time of the fatal injury, were provided by NVDRS as a series of binary variables (i.e., a single case could be coded with multiple circumstances, for example, playing and hunting). To reduce low-count cells in tables and to ease interpretation we created a mutually exclusive hierarchical circumstance variable with four categories: (1) playing > (2) hunting, target shooting, and otherwise operating the firearm with a specific aim in mind > (3) other, and > (4) missing. (Thus, a case in which circumstances included both playing and hunting would be coded as playing).

Injury rates were calculated using population denominators over the years studied drawn from CDC Wonder at the state and county level by gender, single-year age group, race, and ethnicity, with bridged race categories used as racial/ethnic categories for the 2015–2020 data and with single race categories used for the 2021 data as the bridged race data are not available for 2021 (CDC Wonder [11]). Population counts for county types by urbanization were available from 2015 to 2020 through the bridged race dataset. They were not available for the 2021 year through the single race dataset. Since the distribution of racial groups across county types was very stable year-to-year from 2015 to 2020 (not shown), the 2020 racial distribution was used to estimate the county-level population denominators for 2021. Rates were calculated per million children. Incidence rate ratios (IRRs) used White children as the reference group. Statistical significance was not assessed as NVDRS data are a census of relevant deaths for the counties in the sample. JMP 18 and Microsoft Excel were used for analysis.

## Results

Of the 568 UFDs among children 0–17 years of age in our study sample, four-fifths of victims were male (Table 1). Deaths primarily occurred in a home (84%), usually the victim’s home (55%), and involved a child playing with a firearm (63%). Marked variations in incident rates by race were evident across victims’ age and urbanization. For example, 46% of UFDs among Black children were younger than ten, compared with 35% among White children, and whereas rural counties accounted for 8% of UFDs among Black children, they accounted for 42% of UFDs among White children. Among those cases for which gun type was known, handguns were used in the majority of all cases, but long guns were used in 30% of deaths of White victims, compared to only 7% of deaths of Black victims. Other characteristics showed less variation by race. For example, regardless of the victim’s race or ethnicity, at least three-quarters of UFDs occurred in a home, usually the victim’s home. Not shown, data recovered from narrative review accounted for 10% or less of all characteristics, with circumstances having the most recovered data (10%) and urbanicity having the least recovered data (< 1%).

 Nearly half of table cells for our constructed “Other” racial/ethnic group and for the Hispanic racial/ethnic group were suppressed due to data use agreements that necessitated suppression due to low cell counts, precluding further assessment of these characteristics among these subgroups (Table 1). For characteristics we could evaluate among our “Other” racial group, a majority of victims were injured by handguns (66%), injured in a home (77%), and injured while playing (66%) (Table 1). Nearly two thirds of UFDs among Hispanic children (65%) were other-inflicted, the highest proportion among all racial groups. Otherwise, trends in UFDs for Hispanic children mirrored trends for UFDs overall (Table 1).

The rate of UFDs was 4.6 times higher for Black children than for White children, and over 6-fold higher for Black children between 5 and 9 years of age, for Black female, and for Black children living in large central metros where the incident rate was 7.7 times higher than that of White children (Table 2). Rates of UFD among Black children were higher than those of White children in every age group, and highest in Black children 15–17 years of age (11.6 per million). Whereas rates of UFDs for White children increased with county rurality (from approximately 1 to 3 injuries per million), rates among Black children were relatively high through the urban–rural continuum, and were higher than White children’s rates at every level of urbanicity, including the most rural areas.

**Table 1:** Characteristics of unintentional firearm deaths of children (0–17), by race/ethnicity, NVDRS, 2015–2021 (32 states)^1^

Victim Characteristics	Victim Racial Group				
	Black, non-Hispanic	Hispanic	Other	White, non-Hispanic	Total
Total	265 (47%)	51 (9%)	35 (6%)	217 (38%)	568 (100%)
Sex					
Female	53 (20%)	9 (18%)	8 (23%)	32 (15%)	102 (18%)
Male	212 (80%)	42 (82%)	27 (77%)	185 (85%)	466 (82%)
Shooter Identity					
Self-inflicted	111 (42%)	*	*	91 (42%)	231 (41%)
Other-inflicted	137 (52%)	33 (65%)	20 (57%)	107 (49%)	297 (52%)
Family	51 (19%)	9 (18%)	11 (31%)	44 (20%)	115 (20%)
Friend	46 (17%)	17 (33%)	6 (17%)	37 (17%)	106 (19%)
Other	13 (5%)	*	*	7 (3%)	24 (4%)
Missing or Unknown	27 (10%)	*	*	19 (9%)	52 (9%)
Unknown whether self or other-inflicted	17 (6%)	*	*	19 (9%)	40 (7%)
Age					
< 5	79 (30%)	8 (16%)	11 (31%)	53 (24%)	151 (27%)
5–9	43 (16%)	5 (10%)	5 (14%)	23 (11%)	76 (13%)
10–14	55 (21%)	16 (31%)	6 (17%)	57 (26%)	134 (24%)
15–17	88 (33%)	22 (43%)	13 (37%)	84 (39%)	207 (36%)
Death Location					
Home	232 (88%)	45 (88%)	27 (77%)	172 (79%)	476 (84%)
Other Home	78 (29%)	18 (35%)	9 (26%)	58 (27%)	163 (29%)
Victim Home	153 (58%)	27 (53%)	18 (51%)	113 (52%)	311 (55%)
Other	24 (9%)	*	*	*	79 (14%)
Unknown or Missing	9 (3%)	*	*	*	13 (2%)
Firearm Type					
Handgun	196 (74%)	41 (80%)	23 (66%)	136 (63%)	396 (70%)
Long gun	15 (6%)	*	*	59 (27%)	91 (16%)
Missing or Unknown	54 (20%)	*	*	22 (10%)	81 (14%)
County Urbanicity					
Large central metro	94 (35%)	13 (25%)	7 (20%)	21 (10%)	135 (24%)
Large fringe metro	61 (23%)	*	*	38 (18%)	121 (21%)
Medium metro	60 (23%)	10 (20%)	6 (17%)	43 (20%)	119 (21%)
Small metro	29 (11%)	*	*	24 (11%)	60 (11%)
Micropolitan	15 (6%)	*	*	49 (23%)	70 (12%)
Noncore	6 (2%)	5 (10%)	10 (29%)	42 (19%)	63 (11%)
Circumstance**					
Playing	173 (65%)	39 (76%)	23 (66%)	124 (57%)	359 (63%)
H/TS/Other	16 (6%)	*	*	36 (17%)	60 (11%)
Other	42 (16%)	*	*	40 (18%)	91 (16%)
Missing	34 (13%)	*	*	17 (8%)	58 (10%)

1. States included are: Alaska, Arizona, Colorado, Connecticut, Georgia, Kansas, Kentucky, Maine, Maryland, Massachusetts, Michigan, Minnesota, New Hampshire, New Jersey, New Mexico, North Carolina, Ohio, Oklahoma, Oregon, Rhode Island, South Carolina, Utah, Vermont, Virginia, and Wisconsin (2015–2021); Indiana, Iowa (2016–2021); New York (2015–2019, 2021); Washington (2018–2021); Hawaii (2015–2016, 2019); Pennsylvania, Illinois (2020–2021). Select counties from Pennsylvania and Illinois (2016–2019); Washington (2016, 2017)

*Suppressed data

**Playing circumstances include playing, thought toy, and showing; H/TS/Other specific circumstances include hunting, target shooting, cleaning, loading unloading, holstering, firing while engaging safety, and dropping. Other includes other injury mechanism, other context injury, bullet ricochet, self-defense, celebratory firing, unintentionally pulled trigger, and gun defect. It’s further noted no cases of playing were also coded as hunting, and of the 28 deaths associated with hunting or target shooting, White children made up 25 of the decedents

**Table 2:** Rate ratios for unintentional firearm deaths among children (0–17), by race/ethnicity, NVDRS, 2015–2021 (32 states)

Victim Characteristics	Rate Black	Rate White	Rate Ratio Black-White
**Total**	**6.0**	**1.3**	**4.6**
Victim Sex			
Female	2.4	0.4	6.2
Male	9.4	2.2	4.4
Age			
< 5	6.6	1.2	5.4
5–9	3.5	0.5	6.9
10–14	4.4	1.2	3.7
15–17	11.6	2.8	4.2
County Urbanicity			
Large central metro	5.6	0.7	7.7
Large fringe metro	4.7	0.7	6.8
Medium metro	7.2	1.2	6.2
Small metro	8.8	1.4	6.3
Micropolitan and Noncore	6.1	3.0	2.0
Missing	0	0	NA

## Discussion

Prior work using the only source of data validated for coding of unintentional firearm deaths—NVDRS— identified nearly equal counts of UFDs among Black children compared with White children, suggesting a large disparity in the rate of UFD by race [2, 3]. Our study extends these count-based findings by quantifying the Black-White racial disparity in rates per million children and by describing how rate-related disparities vary by select sociodemographic characteristics. Our finding that these disparities are especially large for children between 5 and 9 years of age, for girls, and for children living in urban areas has not been reported previously.

At least two limitations should be kept in mind when interpreting our findings. First, although NVDRS has been validated as an accurate source of information about UFD among children [10], our results are based on data from 32 states and thus may not generalize to the entire United States. Second, while we describe the incidence and toll of UFD among children by race/ethnicity, age, and urbanization, and report on a limited typology of circumstances, the underlying reasons for the disparities we find remain unclear. These disparities may stem from interconnected social, economic, and environmental factors that create differences in access to firearms that children can readily fire. Unfortunately, our study does not provide information about or insight into the prevalence of or interaction between these potential risk factors. Future efforts to augment NVDRS data with information from child fatality review modules (e.g., about adult supervision at the time of the injury) and to improve the completeness of data NVDRS is supposed to collect about the guns involved in these deaths (e.g., who owned the gun, how it was stored) have the potential to better inform policy that aims to mitigate the toll of and racial disparities in these preventable deaths among US children.

## Data Availability

The datasets supporting the conclusions of this article are available in the CDC wonder repository (10.7910/dvn/ua0yge and https://wonder.cdc.gov/) and by request via the NVDRS Restricted Access Database application process: https://www.cdc.gov/nvdrs/about/nvdrs-data-access.html.

## Abbreviations

NCHS: The National Center for Health Statistics
NVDRS: The National Violent Death Reporting System
UFD: Unintentional Firearm Death
